# Promotion of Bone Formation in a Rat Osteoporotic Vertebral Body Defect Model via Suppression of Osteoclastogenesis by Ectopic Embryonic Calvaria Derived Mesenchymal Stem Cells

**DOI:** 10.3390/ijms25158174

**Published:** 2024-07-26

**Authors:** Yerin Yu, Somin Lee, Minsung Bock, Seong Bae An, Hae Eun Shin, Jong Seop Rim, Jun-oh Kwon, Kwang-Sook Park, Inbo Han

**Affiliations:** 1 Department of Neurosurgery, CHA Bundang Medical Center, CHA University, Seongnam-si 13496, Republic of Korea; dhdtm33@naver.com (Y.Y.); dmddmdmd5@gmail.com (S.L.); minsungbock@gmail.com (M.B.); anseongbae@gmail.com (S.B.A.); tlsgodms223@naver.com (H.E.S.); ksp1606@chamc.co.kr (K.-S.P.); 2 Advanced Regenerative Medicine Research Center, CHA Future Medicine Research Institute, Seongnam-si 13488, Republic of Korea; 3 Fetal Stem Cell Research Center, CHA Advanced Research Institute, Seongnam-si 13488, Republic of Korea; rimjs@chamc.co.kr (J.S.R.); jokwon@chamc.co.kr (J.-o.K.)

**Keywords:** osteoporosis, osteoporotic vertebral compression fracture, mesenchymal stem cell, bone regeneration, osteogenesis, ectopic embryonic calvaria derived mesenchymal stem cell

## Abstract

Osteoporotic vertebral compression fractures (OVCFs) are the most prevalent fractures among patients with osteoporosis, leading to severe pain, deformities, and even death. This study explored the use of ectopic embryonic calvaria derived mesenchymal stem cells (EE-cMSCs), which are known for their superior differentiation and proliferation capabilities, as a potential treatment for bone regeneration in OVCFs. We evaluated the impact of EE-cMSCs on osteoclastogenesis in a RAW264.7 cell environment, which was induced by the receptor activator of nuclear factor kappa-beta ligand (RANKL), using cytochemical staining and quantitative real-time PCR. The osteogenic potential of EE-cMSCs was evaluated under various hydrogel conditions. An osteoporotic vertebral body bone defect model was established by inducing osteoporosis in rats through bilateral ovariectomy and creating defects in their coccygeal vertebral bodies. The effects of EE-cMSCs were examined using micro-computed tomography (μCT) and histology, including immunohistochemical analyses. In vitro, EE-cMSCs inhibited osteoclast differentiation and promoted osteogenesis in a 3D cell culture environment using fibrin hydrogel. Moreover, μCT and histological staining demonstrated increased new bone formation in the group treated with EE-cMSCs and fibrin. Immunostaining showed reduced osteoclast activity and bone resorption, alongside increased angiogenesis. Thus, EE-cMSCs can effectively promote bone regeneration and may represent a promising therapeutic approach for treating OVCFs.

## 1. Introduction

Osteoporotic vertebral compression fractures (OVCFs) represent the most prevalent form of osteoporotic fractures, impacting around 1.4 million individuals globally each year [1]. Severe OVCFs can lead to intense back pain, spinal deformity, decreased mobility, diminished pulmonary function, and heightened age-adjusted mortality risk, significantly compromising the quality of life for those affected [2]. Consequently, there is an urgent need to develop and refine existing treatment strategies to manage OVCFs more effectively and improve patient outcomes.

Available treatments for OVCFs primarily focus on managing pain, preventing further bone loss, and employing invasive procedures that fail to address the underlying causes. This highlights the urgent need for research into more effective treatment options. Conservative treatments, such as nonsteroidal anti-inflammatory drugs (NSAIDs), help alleviate pain but do not treat the osteoporosis itself [3,4]. Common osteoporotic medications, including antiresorptive agents like bisphosphonates and denosumab, as well as anabolic agents such as teriparatide and romosozumab, have demonstrated limited effectiveness in promoting bone healing [5]. Percutaneous kyphoplasty and percutaneous vertebroplasty use polymethylmethacrylate (PMMA) to treat OVCFs. While this invasive procedure provides temporary pain relief and rigid structural support to stabilize microfractures through an exothermic reaction, it has notable drawbacks. PMMA releases toxic monomers that can increase cell death in the surrounding tissue. Additionally, these procedures carry significant risks, including cement leakage and adjacent segmental fractures, and they do not address the underlying osteoporotic condition [6]. Furthermore, the long-term benefits are questionable, as evidenced by randomized controlled trials showing no significant long-term advantages [7,8,9,10,11].

Previous research has shown that mesenchymal stem cell (MSC) transplantation in vivo can increase bone mineral density (BMD), enhance trabecular bone volume, and repair osteoporotic bone damage [12,13,14]. A recent clinical trial reported that the combination of umbilical cord Wharton’s jelly-derived mesenchymal stem cells and teriparatide significantly improved pain and bone density compared to the group that received only teriparatide [15]. Just as umbilical cord Wharton’s jelly-derived MSCs have superior proliferation and immune-regulatory abilities compared to MSCs obtained from adults, fetal-derived MSCs also exhibit enhanced capabilities [16]. These fetal MSCs are known for their superior proliferation, survival, immune-regulatory abilities, and inflammation suppression compared to adult-derived stem cells. Fetal MSCs can be derived from various tissues, such as bone marrow, kidney, muscle, dermis, placenta, and amniotic fluid, making them useful for application. Notably, our fetal MSCs were isolated from the fetal skull and henceforth referred to as “Human ectopic embryonic calvaria derived mesenchymal stem cells (EE-cMSCs)” [17]. Previous research has shown that EE-cMSCs demonstrate statistically significant superior osteogenic differentiation potential and higher osteogenic gene expression, leading to increased bone formation capacity compared to adult-derived bone marrow mesenchymal stem cells (BM-MSCs) (Figure S1) and UC-MSCs [18,19,20,21,22,23]. Given the demonstrated beneficial effects of MSCs on osteoporosis, EE-cMSCs, with their enhanced differentiation and proliferation abilities, hold significant potential as a treatment for bone regeneration in OVCFs.

In this study, we induced the characteristics of OVCFs in rats through bilateral ovariectomy and subsequent creation of a bone defect in the coccygeal vertebral body [24,25,26,27]. We investigated the effects of treatment with EE-cMSCs and fibrin gel compared to an untreated control group, with a focus on new bone formation in the defect area of osteoporotic vertebral bones. The progression of bone healing was sequentially evaluated using micro-computed tomography (µCT) imaging and histological analysis [28].

## 2. Results

### 2.1. EE-cMSCs Inhibit RANKL-Induced Osteoclast Differentiation

First, we investigated the inhibitory impact of EE-cMSCs on the differentiation of RAW264.7 murine macrophages (hereafter referred to as RAW cells) into osteoclasts. The RAW cells were treated with the receptor activator of nuclear factor kappa-beta ligand (RANKL) to induce osteoclast differentiation.

**Figure 1:**
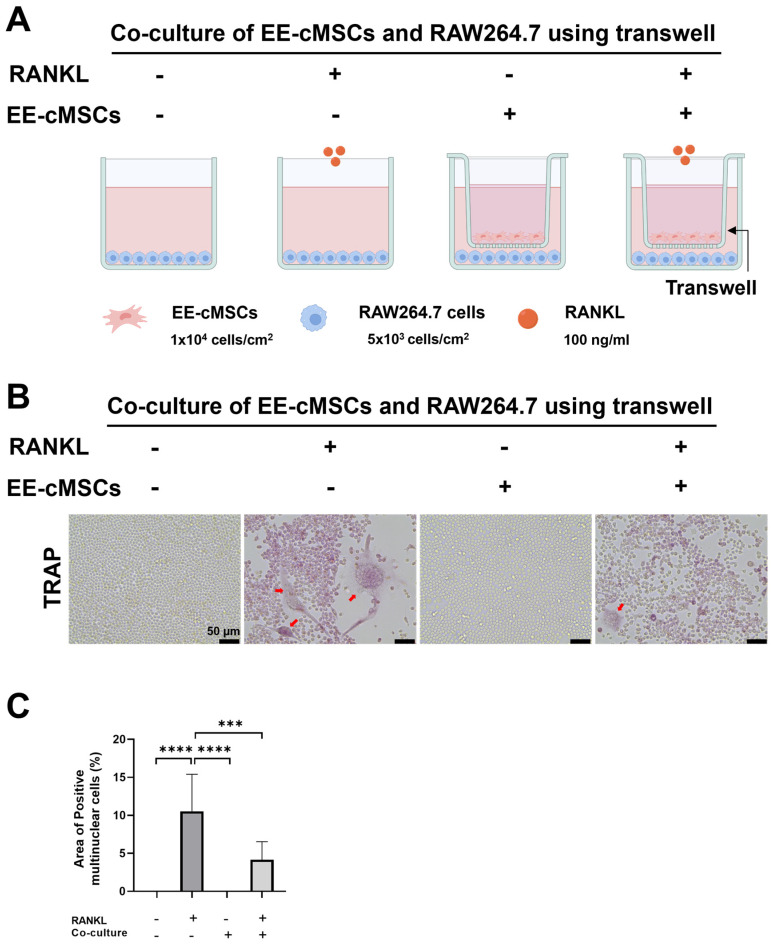

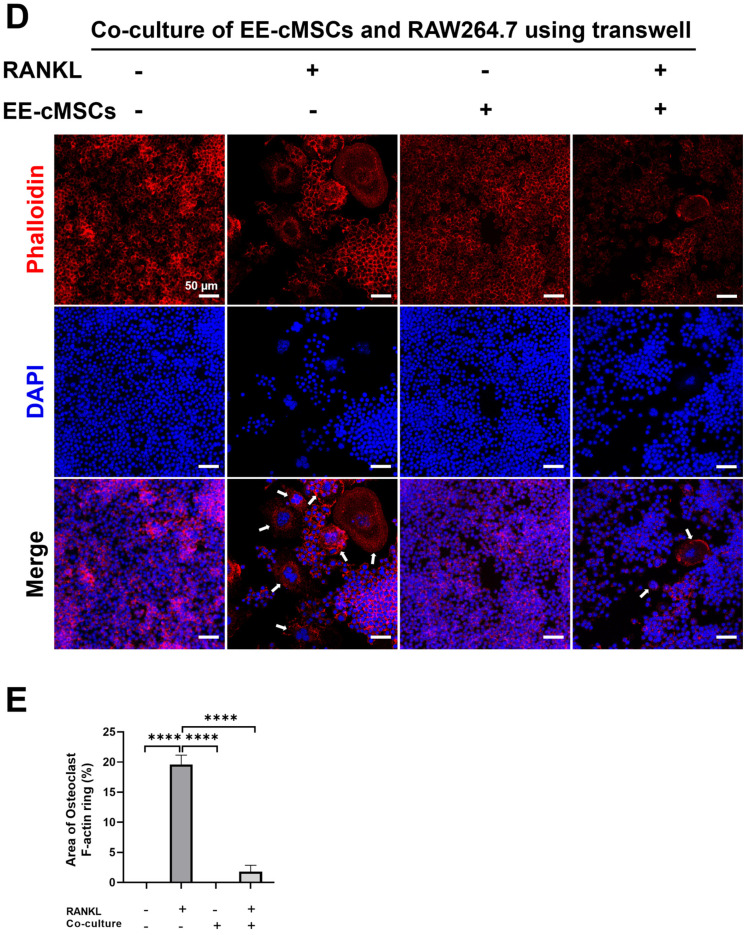

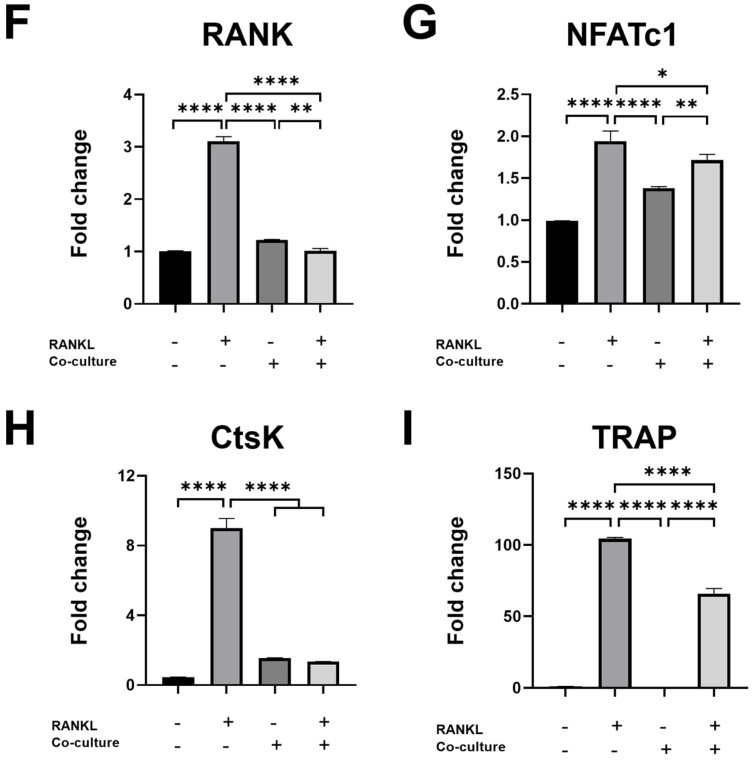
Inhibitory effect of EE-cMSCs on osteoclast differentiation in co-culture with RAW264.7 cells. (**A**) Schematic diagram of the Transwell co-culture system. (**B**) TRAP staining of osteoclasts derived from RAW264.7 cells in the Transwell culture system. (**C**) Quantitative analysis showing the area of TRAP-positive multinucleated cells. (**D**) Phalloidin staining of osteoclast actin ring formation in the Transwell culture system. (**E**) Quantitative analysis showing the area of osteoclast F-actin rings. Gene expression analyses of (**F**) receptor activator of nuclear factor kappa-B (RANK), (**G**) nuclear factor of activated T-cells, cytoplasmic 1 (NFATc1), (**H**) cathepsin K (CtsK), and (**I**) tartrate-resistant acid phosphatase (TRAP) in the Transwell groups after 4 days of co-culture. Statistical significance for quantitative analyses was assessed using one-way ANOVA, followed by Tukey’s correction. * *p* < 0.05; ** *p* < 0.01; *** *p* < 0.001; **** *p* < 0.0001. All data are expressed as mean ± standard deviation (SD). Figure created with BioRender.com (accessed on 9 April 2024).

As shown in Figure 1A, we conducted a co-culture of EE-cMSCs and RAW cells using a Transwell system. The experimental setup included the following four distinct groups: 1. Experimental Group 1 (RANKL+, EE-cMSC-): This group was treated with 100 ng/mL RANKL to induce osteoclast differentiation in the RAW cells without the presence of EE-cMSCs. 2. Experimental Group 2 (RANKL+, EE-cMSC+): This group was treated with 100 ng/mL RANKL and co-cultured with EE-cMSCs to evaluate the impact of EE-cMSCs on RANKL-induced osteoclast differentiation. 3. Negative Control Group (RANKL-, EE-cMSC-): This group did not receive RANKL treatment and was not co-cultured with EE-cMSCs, serving as a baseline to observe the natural state of the RAW cells without osteoclast induction or EE-cMSC influence. 4. Positive Control Group (RANKL-, EE-cMSC+): This group did not receive RANKL treatment but was co-cultured with EE-cMSCs to assess any potential effects of EE-cMSCs on the RAW cells in the absence of osteoclast differentiation signals. Following the administration of RANKL, the differentiation of the RAW cells into mature, multinucleated osteoclasts was observed over a period of 4 days [29].

Tartrate-resistant acid phosphatase (TRAP) staining was used to confirm the formation of mature multinucleated osteoclasts [30]. In the group treated with RANKL alone (RANKL+, EE-cMSC-), there was an increased formation of large multinucleated and TRAP-positive cells (10.55 ± 4.864%) (Figure 1B,C). Conversely, the co-culture group with RANKL-treated EE-cMSCs (RANKL+, EE-cMSC+) demonstrated a significant suppression in the development of mature multinucleated osteoclasts (4.177 ± 2.357%; *p* < 0.0001) (Figure 1B,C). These findings indicate that EE-cMSCs inhibit RANKL-induced osteoclastogenesis.

The organization of the cytoskeleton is crucial for osteoclast differentiation as it plays a vital role in the formation of actin rings, which are hallmark structures of mature osteoclasts [31,32]. To confirm the organization of the cytoskeleton, we utilized phalloidin staining. In the RANKL+, EE-cMSC- group, the cells gradually fused to form large, multinucleated cells, displaying complete F-actin rings (19.62 ± 1.539%) (Figure 1D,E). However, in the RANKL+, EE-cMSC+ group, the F-actin rings were incomplete, and the fused cells were smaller (1.833 ± 1.042%; *p* < 0.0001) (Figure 1D,E). Consequently, the RANKL+, EE-cMSC+ group significantly inhibited the differentiation of RAW cells into osteoclasts.

To investigate the molecular mechanisms behind the inhibition of osteoclast differentiation by EE-cMSCs, we analyzed the expression of various genes. These genes included those encoding the transcription factor NFATc1, which regulates osteoclastogenesis, as well as enzymatic proteins specific to multinucleated osteoclasts, such as TRAP and CtsK, and the receptor involved in osteoclast differentiation, RANK [33].

In the cells induced by RANKL (RANKL+, EE-cMSC-), the expression of RANK was upregulated (3.107 ± 0.08681%). Conversely, the RANKL+, EE-cMSC+ group significantly inhibited osteoclast differentiation (1.014 ± 0.0456%; *p* < 0.0001) (Figure 1F). Additionally, in the RANKL+, EE-cMSC- group, there was an upregulation in the expression of NFATc1, CtsK, and TRAP (1.943 ± 0.1197%, 9.004 ± 0.5558%, and 104.5 ± 0.8975%, respectively). However, the introduction of EE-cMSCs (RANKL+, EE-cMSC+) significantly reduced the expression of these markers (1.715 ± 0.0702%, 1.349 ± 0.003466%, and 65.93 ± 3.638%, respectively) (Figure 1G–I).

### 2.2. EE-cMSCs Exhibit Osteogenic Effects in Fibrin Gel

**Figure 2:**
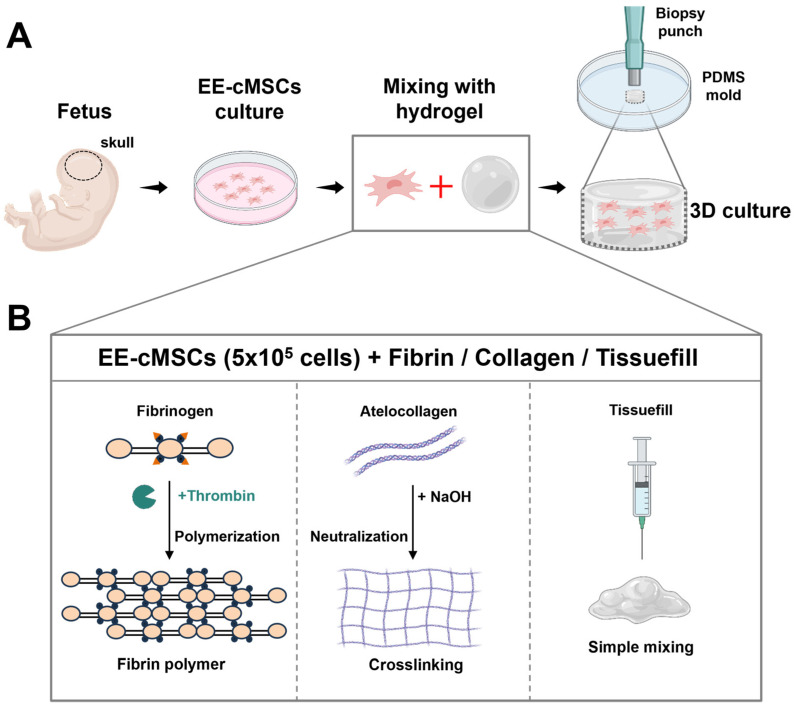

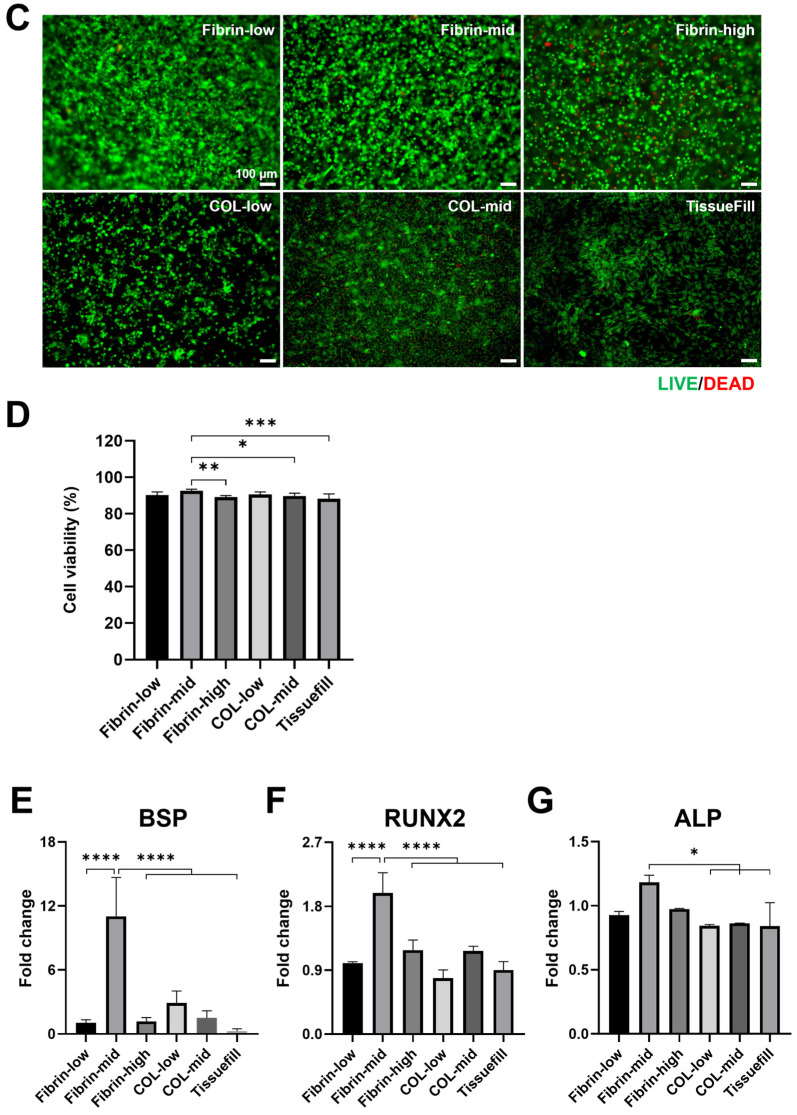
Preparation and evaluation of the osteogenic potential of EE-cMSCs in various hydrogels for cell delivery. (**A**) Schematic illustration of EE-cMSC culture and three-dimensional bone formation. (**B**) Application of fibrin, collagen, and TissueFill in 3D cell culture. (**C**) Live/Dead staining of EE-cMSCs with low, mid, and high concentrations of fibrin and collagen, as well as TissueFill. (**D**) Viability (%) of EE-cMSCs based on live/dead image analysis. (**E**) Gene expression analyses of bone sialoprotein (BSP), (**F**) runt-related transcription factor 2 (RUNX2), and (**G**) alkaline phosphatase (ALP) in different hydrogel groups after 2 weeks. Statistical significance for quantitative analyses was assessed using one-way ANOVA, followed by Tukey’s correction. * *p* < 0.05; ** *p* < 0.01; *** *p* < 0.001; **** *p* < 0.0001. All data are expressed as mean ± standard deviation (SD). Figure created with BioRender.com (accessed on 9 April 2024).

We conducted 3D cell cultures to investigate the cytocompatibility, cytotoxicity, and osteogenic effects of EE-cMSCs under various injectable hydrogel conditions, including those containing bone morphogenetic protein-2 (BMP-2) at a concentration of 5 μg/mL (Figure 2A,B) [34,35,36]. As shown in Table 1, EE-cMSCs were subjected to various concentrations within the fibrin and collagen groups, as well as the TissueFill group.

The live/dead analysis results indicated that cell viability was approximately 90% after 24 h of culture across various groups as follows: fibrin-low, fibrin-mid, fibrin-high, collagen-low, collagen-mid, and TissueFill, with viabilities of 90.3 ± 1.616%, 92.66 ± 0.7975%, 89.01 ± 0.9618%, 90.56 ± 1.408%, 89.76 ± 1.481%, and 88.25 ± 2.563%, respectively (Figure 2C,D). The fibrin-mid concentration demonstrated relatively higher cell viability compared to the fibrin-high, collagen-mid, and TissueFill concentrations (*p* < 0.01, *p* < 0.05, and *p* < 0.001, respectively; Figure 2D).

Gene expression was analyzed to assess the osteogenic effect of EE-cMSCs under various hydrogel concentration conditions [37,38]. On day 14 of the 3D cell culture, we measured the expression of genes associated with bone formation in the fibrin, collagen, and TissueFill groups. The expression of bone sialoprotein (BSP), an early marker of osteogenic differentiation, was significantly higher in the fibrin-mid group (11.03 ± 3.646%) compared to the other groups (*p* < 0.0001, Figure 2E). Similarly, the expression levels of runt-related transcription factor 2 (RUNX2), another early marker, were elevated in the fibrin-mid group (1.986 ± 0.2847%) relative to other groups (*p* < 0.0001, Figure 2F). Additionally, the expression levels of alkaline phosphatase (ALP), also an early marker, were higher in the fibrin-mid group (1.181 ± 0.05726%) compared to other groups (Figure 2F). In summary, EE-cMSCs exhibited the most significant osteogenic effect under fibrin-mid concentration conditions.

### 2.3. EE-cMSCs with Fibrin Gel Transplantation Promote Bone Regeneration in an Osteoporotic Vertebral Body Bone Defect Model

**Figure 3:**
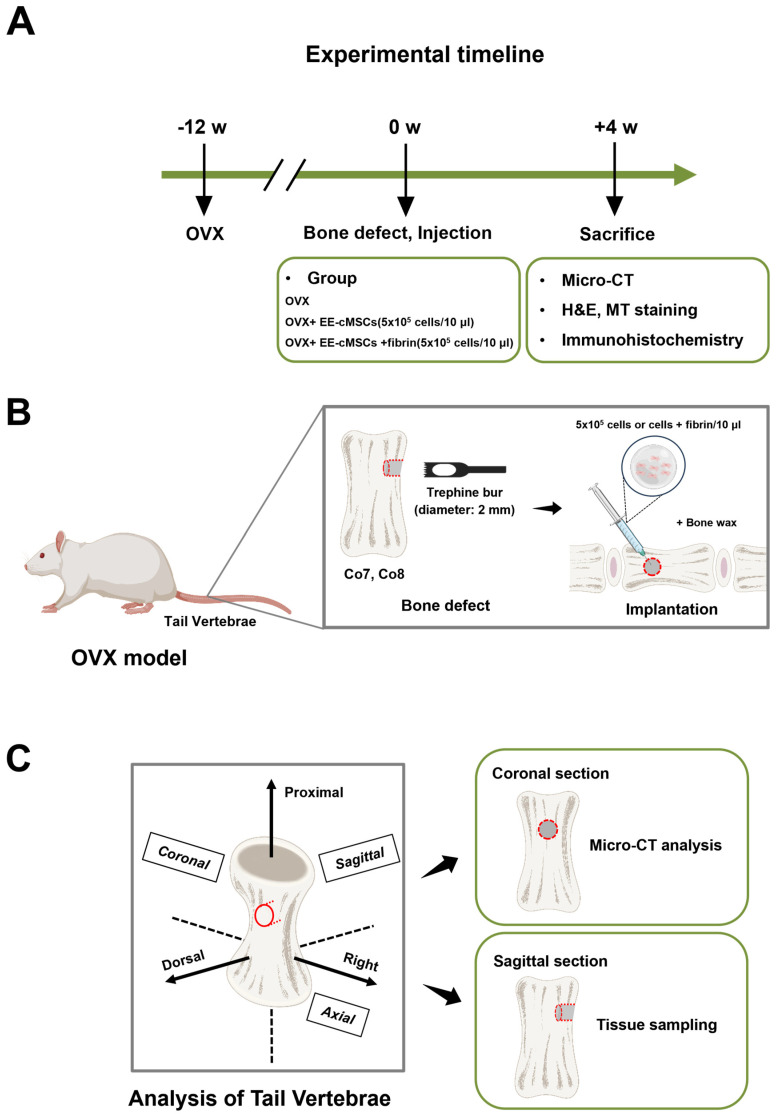
Experimental design and procedure for evaluating EE-cMSCs in an OVCF rat model. (**A**) Experimental timeline for EE-cMSCs and EE-cMSCs with fibrin implantation, followed by evaluation after osteoporotic vertebral compression fractures (OVCFs). (**B**) Illustration of the method used to model OVCFs for assessing the therapeutic efficacy of prepared EE-cMSCs and EE-cMSCs with fibrin. (**C**) Description of the rat tail vertebral defect site in the OVCF model, including the orientation for analysis. Figure created with BioRender.com (accessed on 9 April 2024).

To evaluate the therapeutic potential of EE-cMSCs for bone regeneration, we administered them in a rat model with an osteoporotic vertebral body defect. We compared the effects of EE-cMSCs alone to those of EE-cMSCs combined with fibrin implantation (Figure 3A,B).

**Figure 4:**
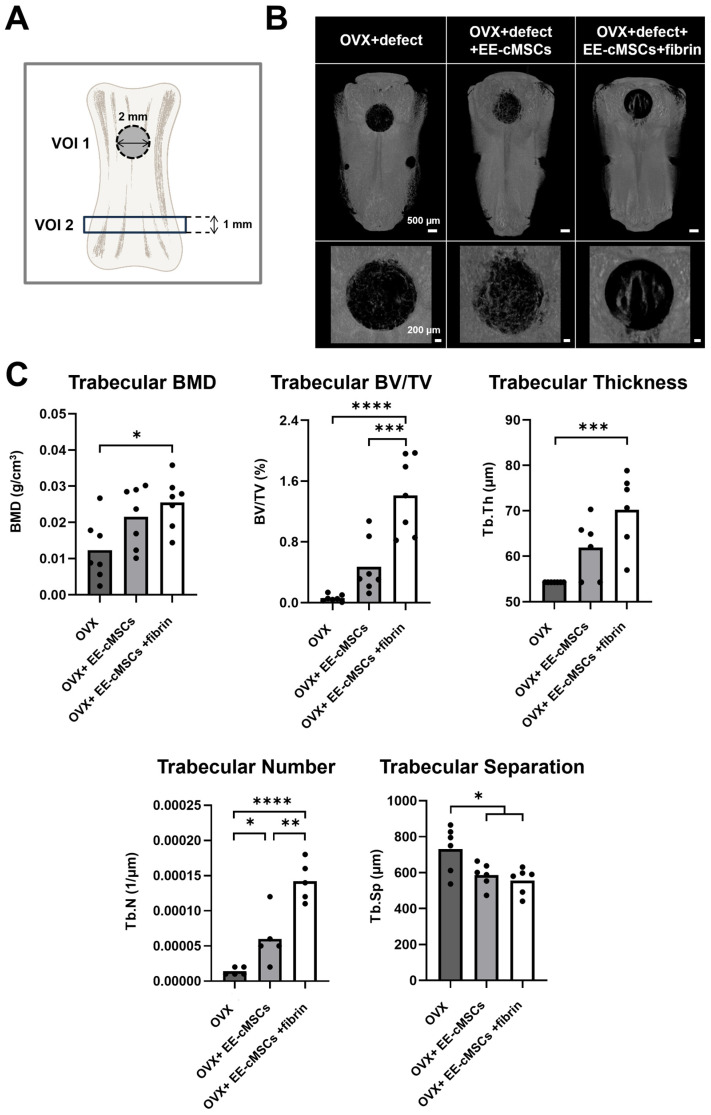

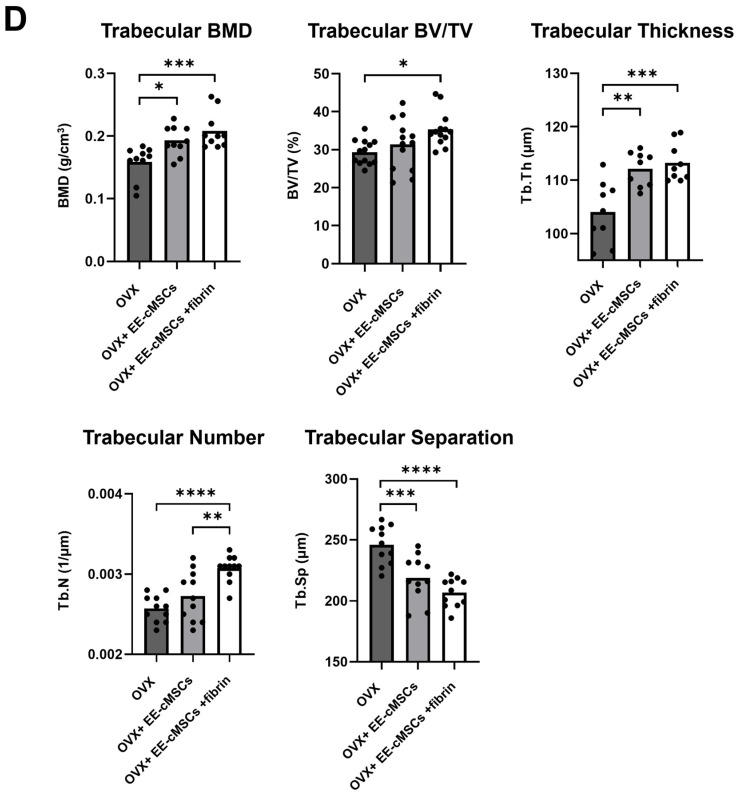
Micro-computed tomography (μCT) evaluation and morphometric analysis of bone formation in bone defects 4 weeks after implantation of EE-cMSCs and EE-cMSCs with fibrin. (**A**) Schematic illustration of the volume of interest (VOI) for micro-CT analysis. VOI 1: 2 mm diameter defect region; VOI 2: 1 mm height selected for the analysis of trabecular bone. (**B**) Three-dimensional reconstruction images of the tail vertebral measured by micro-CT at 4 weeks after osteoporotic vertebral compression fracture (OVCF). (**C**) Bone defect site and (**D**) Trabecular bone site showing bone mineral density (BMD, g/cm^3^), trabecular separation (Tb.Sp, mm), trabecular thickness (Tb.Th, mm), trabecular number (Tb.N, 1/mm), and the ratio of bone volume (BV) to total volume (TV) (BV/TV, %). Statistical significance for quantitative analyses was assessed using one-way ANOVA, followed by Tukey’s correction. * *p* < 0.05; ** *p* < 0.01; *** *p* < 0.001; **** *p* < 0.0001. All data are expressed as mean ± standard deviation (SD).

Four weeks post-transplantation, µCT analysis was conducted on the defect area (volume of interest [VOI] 1) (Figure 3C) and the trabecular bone area (VOI 2) (Figure 4A) [39]. In the 3D µCT images shown in Figure 4B, the EE-cMSC group displayed new bone formation primarily at the edges of the defect area. In contrast, the EE-cMSCs with fibrin group showed new bone formation extending from the periphery toward the center of the defect, compared to the ovariectomy (OVX) group [40]. We performed a comprehensive analysis of various bone microstructure parameters in both VOI 1 and VOI 2, including bone mineral density (BMD), bone volume over total volume (BV/TV), trabecular thickness (Tb.Th), trabecular number (Tb.N), and trabecular separation (Tb.Sp).

In VOI 1, the EE-cMSCs with fibrin group demonstrated a significantly higher BMD of 0.02735 ± 0.005542 g/cm^3^ than the OVX group (0.0099 ± 0.006001 g/cm^3^; *p* < 0.01) and the EE-cMSC group (0.02008 ± 0.008171 g/cm^3^), as shown in Figure 4C. Additionally, the quantification of newly formed bone volume relative to total volume in VOI 1 indicated that the EE-cMSCs with fibrin group (1.409 ± 0.5075%) had a significantly higher BV/TV than the OVX group (0.059 ± 0.04399%; *p* < 0.0001) and the EE-cMSC group (0.4684 ± 0.3588%; *p* < 0.001) (Figure 4C). The analysis of trabecular bone thickness in VOI 1 revealed that the EE-cMSCs with fibrin group (70.24 ± 8.249 µm) exhibited a significantly higher Tb.Th than the OVX group (54.27 µm; *p* < 0.001). However, this measurement did not significantly differ from that of the EE-cMSC group (61.93 ± 6.492 µm) (Figure 4C.) The trabecular number in VOI 1 also showed that the EE-cMSCs with fibrin group (0.000142 ± 0.00002864 1/µm) had a significantly higher Tb.N than both the EE-cMSC group (0.00006 ± 0.00003674 1/µm; *p* < 0.01) and the OVX group (0.000014 ± 0.000005477 1/µm; *p* < 0.0001) (Figure 4C). Furthermore, trabecular separation in VOI 1 was highest in the OVX group (731.2 ± 129.1 µm). In contrast, both the EE-cMSC group (586.9 ± 67.47 µm; *p* < 0.05) and the EE-cMSCs with fibrin group (555.5 ± 72.76 µm; *p* < 0.05) exhibited lower measurements, indicating decreased separation (Figure 4C).

Additionally, to investigate the impact of EE-cMSCs transplantation on trabecular bone remodeling around the defect area, we analyzed a 1 mm thick section of VOI 2. The analysis revealed that the EE-cMSC group (0.1931 ± 0.02316 g/cm^3^; *p* < 0.05) and the EE-cMSCs with fibrin group (0.2085 ± 0.02910 g/cm^3^; *p* < 0.001) exhibited significantly higher BMD values compared to the OVX group (0.1588 ± 0.02622 g/cm^3^) (Figure 4D). Additionally, the BV/TV in VOI 2 indicated that the EE-cMSCs with fibrin group (35.37 ± 4.572%) had an increased BV/TV compared to the OVX group (29.35 ± 3.232%; *p* < 0.05), although it did not significantly differ from the EE-cMSC group (31.40 ± 6.645%) (Figure 4D).

Analysis of Tb.Th in VOI 2 showed that both the EE-cMSC group (112.1 ± 3.215 µm; *p* < 0.01) and the EE-cMSCs with fibrin group (113.3 ± 3.564 µm *p* < 0.001) had significantly greater thickness than the OVX group (104.1 ± 5.748 μm) (Figure 4D). Additionally, the EE-cMSCs with fibrin group demonstrated a significantly higher trabecular number (Tb.N) (0.003073 ± 0.0001618 1/µm) than both the EE-cMSC group (0.002727 ± 0.0003101 1/µm *p* < 0.01) and the OVX group (0.002573 ± 0.0001679 1/µm; *p* < 0.0001) (Figure 4D). Trabecular separation in VOI 2 was also significantly higher in the OVX group (246.0 ± 15.73 µm), whereas it was reduced in the EE-cMSC group (218.9 ± 18.58 µm *p* < 0.001) and further decreased in the EE-cMSCs with fibrin group (206.8 ± 11.69 µm *p* < 0.0001) (Figure 4D).

**Figure 5:**
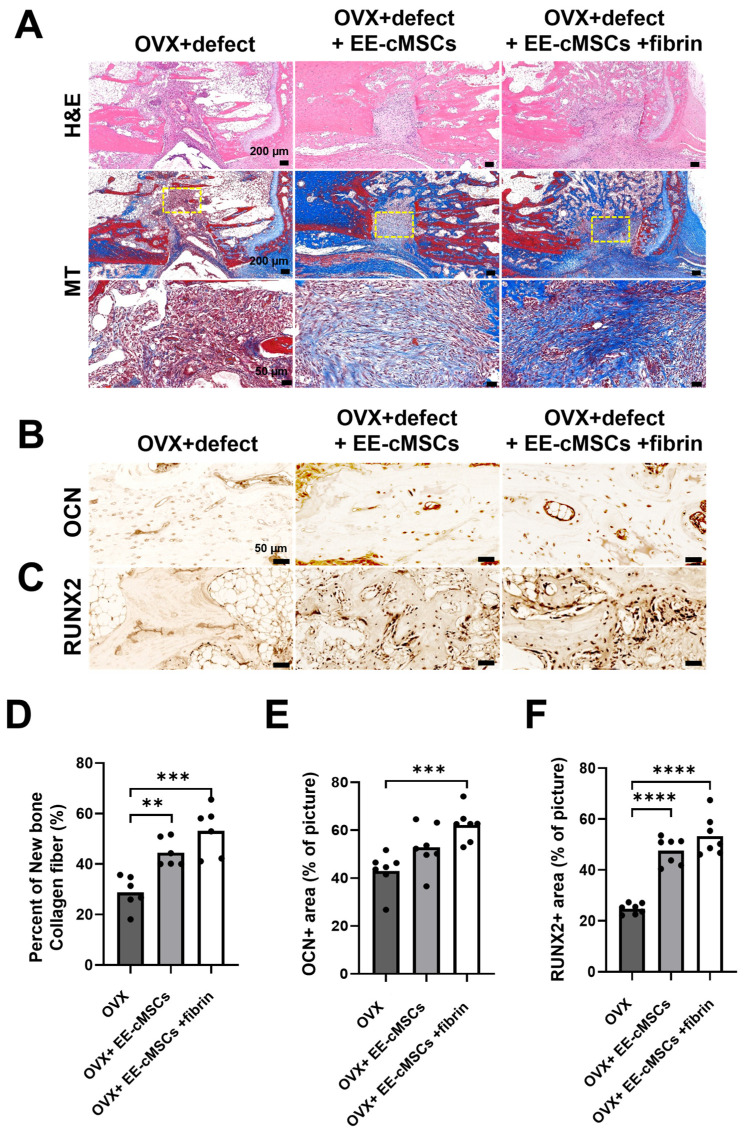
Enhanced bone regeneration by EE-cMSCs and EE-cMSCs with fibrin at 4 weeks after implantation. (**A**) Representative images of tail vertebral sagittal sections stained with Masson trichrome (MT) and hematoxylin and eosin (H&E) at 4 weeks after osteoporotic vertebral compression fracture (OVCF) across three experimental groups. (**B**) Immunohistochemical staining for osteocalcin (OCN)-positive protein expression and (**C**) runt-related transcription factor 2 (RUNX2)-positive protein expression, both indicative of osteogenesis in the trabecular bone. (**D**) Quantitative analysis of the new bone collagen fiber area (blue) from MT-stained images. Quantitative analyses of OCN-positive (OCN+) protein (**E**) and RUNX2-positive (RUNX2+) protein (**F**) expression across different groups. Statistical significance for quantitative analyses was assessed using one-way ANOVA, followed by Tukey’s correction. ** *p* < 0.01; *** *p* < 0.001; **** *p* < 0.0001. All data are expressed as mean ± standard deviation (SD).

To evaluate the effects of EE-cMSCs and EE-cMSCs with fibrin on new bone formation, we conducted hematoxylin and eosin (H&E), Masson trichrome (MT), and immunohistochemistry staining on the bone tissue sections harvested four weeks post-transplantation (Figure 3C). H&E staining revealed that the EE-cMSC groups had more trabecular bone and less trabecular space compared to the OVX group, aligning with the µCT data (Figure 5A). MT staining displayed the new bone collagen fibers in blue and the mature bone in red [41]. The EE-cMSC group (44.53 ± 5.505%; *p* < 0.01) and the EE-cMSCs with fibrin group (53.14 ± 9.761%; *p* < 0.001) showed significantly higher amounts of new bone collagen fibers than the OVX group (28.79 ± 6.587%) (Figure 5A,D).

To further evaluate bone regeneration at the site of osteoporotic vertebral bone defects, we employed osteocalcin (OCN) and RUNX2 immunohistochemistry staining (Figure 5B,C). Osteocalcin, which targets a non-collagen protein secreted by osteoblasts, was used to assess osteogenesis [42]. The EE-cMSCs with fibrin group (62.09 ± 6.919%) demonstrated significantly higher osteogenesis, as indicated by OCN expression, than the OVX group (42.99 ± 7.812%; *p* < 0.01) (Figure 5B,E). However, there was no significant difference in osteogenesis between the OVX group and the EE-cMSC group (52.87 ± 9.362%) (Figure 5B,E). Additionally, we conducted immunostaining for RUNX2, a transcription factor critical for osteoblast differentiation, to further evaluate osteogenesis. Both the EE-cMSC group (47.55 ± 5.341%; *p* < 0.0001) and the EE-cMSCs with fibrin group (53.32 ± 7.759%; *p* < 0.0001) showed significantly higher levels of RUNX2 expression than the OVX group (24.77 ± 2.056%) (Figure 5C,F).

### 2.4. EE-cMSCs with Fibrin Gel Transplantation Regulate Bone Remodeling Post-Osteoporotic Vertebral Bone Defect

To investigate the bone remodeling response, we performed immunohistochemistry staining for RANKL and osteoprotegerin (OPG), which are critical cytokines produced by bone-forming cells. RANKL is a marker for osteoclast differentiation from bone marrow macrophage precursors, while OPG serves as an inhibitor of osteoclast formation. This method enabled us to examine the regulation of bone remodeling by assessing changes in these cytokines within the bone tissue microenvironment of OVX rats following EE-cMSCs with fibrin implantation [43]. Our analysis showed that the EE-cMSC group (58.12 ± 8.484%; *p* < 0.0001) and the EE-cMSCs with fibrin group (59.02 ± 7.787%; *p* < 0.0001) demonstrated significantly higher OPG expression around the bone defect site compared to the OVX group (25.20 ± 2.295%) (Figure 5A,D). Conversely, the OVX group (29.67 ± 5.398%) exhibited significantly higher RANKL expression compared to both the EE-cMSC group (14.42 ± 5.448%; *p* < 0.0001) and the EE-cMSCs with fibrin group (9.137 ± 3.695%; *p* < 0.0001) (Figure 5B,E). These findings indicate that EE-cMSCs with fibrin implantation modulate the RANKL and OPG signaling pathways in osteogenic cells, thereby inhibiting osteoclast formation and bone resorption in the trabecular bone microenvironment.

### 2.5. EE-cMSCs with Fibrin Gel Transplantation Promote Angiogenesis Post-Osteoporotic Vertebral Bone Defect

**Figure 6:**
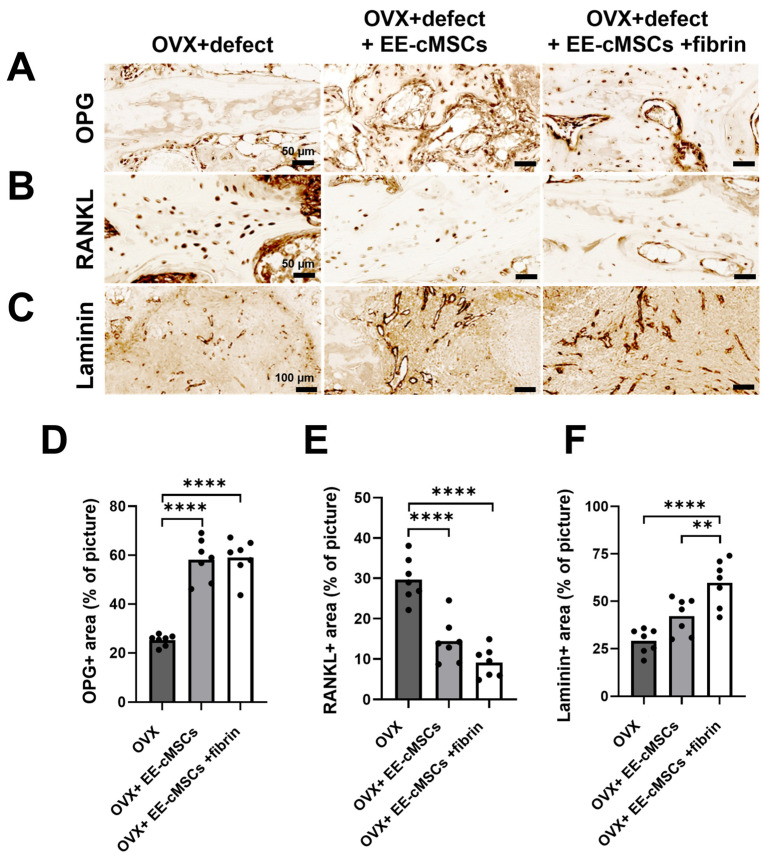
Inhibition of excessive osteoclastogenic activity, bone resorption, and angiogenesis by EE-cMSCs and EE-cMSCs with fibrin at 4 weeks post-implantation. This figure shows the expression of osteoprotegerin (OPG)-positive protein (**A**) and receptor activator of nuclear factor kappa-B ligand (RANKL)-positive protein (**B**), both indicative of bone remodeling in the trabecular bone at 4 weeks after osteoporotic vertebral compression fracture (OVCF). Additionally, it includes the expression of Laminin-positive protein around the defect site at the same time point (**C**). Quantitative results of OPG-positive (OPG+) protein (**D**), RANKL-positive (RANKL+) protein (**E**), and Laminin-positive (laminin+) protein (**F**) expression across different groups are also presented. Statistical significance for quantitative analyses was assessed using one-way ANOVA, followed by Tukey’s correction. ** *p* < 0.01; **** *p* < 0.0001. All data are expressed as mean ± standard deviation (SD).

To assess vascularization at the site of osteoporotic vertebral body defect lesions following EE-cMSCs implantation, we conducted immunohistochemistry using laminin, a marker indicative of the basement membrane involved in vascular structure formation [44]. Laminin expression, which delineates the basement membranes of vessels, was significantly higher in the EE-cMSCs with fibrin group (59.80 ± 12.26%) compared to the OVX group (29.10 ± 6.491%; *p* < 0.0001) and the EE-cMSC group (42.25 ± 9.552%; *p* < 0.01) (Figure 6C,F). These results indicate that implantation of EE-cMSCs with fibrin significantly promotes angiogenesis.

## 3. Discussion

In this study, we observed that EE-cMSCs inhibit osteoclast differentiation and exhibit strong osteogenic effects when combined with fibrin gel. Using a rat model with osteoporotic vertebral body bone defects, we found that the implantation of EE-cMSCs with fibrin effectively suppressed bone resorption and significantly improved bone formation capabilities. Additionally, EE-cMSCs with fibrin implantation demonstrated the potential to promote bone regeneration through the formation of vascular structures.

**Figure 7:**
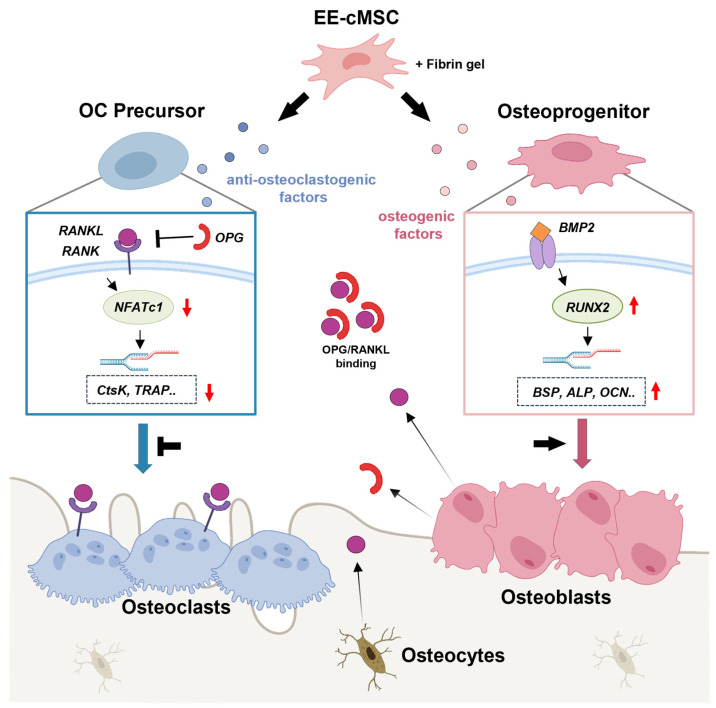
Illustration of the impact of EE-cMSCs with fibrin on the inhibition of osteoclast differentiation and promotion of osteogenesis. After tail vertebrae implantation, EE-cMSCs with fibrin secrete OPG, which competitively inhibits the RANKL–RANK interaction. This inhibition curtails osteoclast differentiation in bone defects. Furthermore, EE-cMSCs with fibrin also suppress the activation of NFATc1, a critical transcription factor in osteoclast formation, thereby reducing the expression of osteoclast-specific genes such as CtsK and TRAP. Conversely, EE-cMSCs with fibrin stimulate RUNX2, an essential transcription factor for osteoblast differentiation. This stimulation leads to the increased expression of osteoblast-specific genes including BSP, ALP, and OCN, which in turn increases osteoblast activity and promotes bone formation.

RAW cells are a murine macrophage cell line that can differentiate into osteoclast-like cells in response to RANKL stimulation, making them a widely used model for studying osteoclastogenesis [29,45,46,47,48]. Our in vitro experiments demonstrated that EE-cMSCs significantly inhibited the differentiation of RAW cells into osteoclasts in a RANKL-induced environment. This inhibition was evidenced by a marked reduction in the formation of TRAP-positive multinucleated cells and the presence of incomplete F-actin rings [48,49,50], observed in both the Transwell system (Figure 1A–E) and in conditioned medium co-culture with EE-cMSCs (Figure S2). The differentiation of RAW cells into osteoclasts is crucial for understanding the osteoclastogenesis inhibitory effects of EE-cMSCs. By inhibiting osteoclastogenesis, EE-cMSCs can prevent excessive bone loss and promote bone regeneration, highlighting their therapeutic potential in treating OVCFs. At the molecular level, there was a significant downregulation in the expression of osteoclastogenic genes such as RANK, NFATc1, CtsK, and TRAP in the presence of EE-cMSCs. RANKL interacts with RANK on osteoclast precursors to initiate osteoclast differentiation. This interaction includes the trimerization of tumor necrosis factor receptor (TNFR)-associated factor 6 (TRAF6) and RANK, which triggers downstream signaling, including the activation of NFATc1. RANKL-mediated NFATc1 signaling activates genes such as Ctsk and TRAP, promoting the terminal differentiation of osteoclasts [51]. Thus, we demonstrated that EE-cMSCs can inhibit osteoclast differentiation in the RANKL-induced osteoclastogenesis process by suppressing the interaction between RANKL and RANK, accompanied by reduced mRNA expression of various osteoclast-specific genes such as NFATc1, CtsK, and TRAP. We confirmed that the application of EE-cMSCs with fibrin inhibits the formation and activity of osteoclasts in the bone defect areas. Bone remodeling is a continuous process of bone formation and resorption, where the balance between RANKL and OPG is crucial. RANKL promotes the differentiation and activation of osteoclasts, while OPG binds to RANKL, preventing its interaction with RANK and thereby inhibiting RANKL signaling. When this balance is disrupted, it can lead to excessive bone resorption [52,53,54,55]. In this study, we observed a significant suppression of RANKL and an increase in OPG expression following the implantation of EE-cMSCs with fibrin. This regulation of bone metabolism suggests an improvement in bone remodeling and healing (Figure 7). Furthermore, Supplementary Table S2 provides a comprehensive literature review on current trends in molecular biological research on osteogenesis and osteoclastogenesis inhibition mechanisms. This table summarizes key studies on the role of the RANKL pathway and the therapeutic potential of targeting osteoclastogenesis [48,49,56,57,58,59,60,61,62,63,64,65].

The osteogenic potential of EE-cMSCs was notably observed in the 3D culture of fibrin gel. Fibrin, known for its excellent biocompatibility, serves as an ideal natural scaffold material for supporting MSCs in bone tissue engineering [66]. Our study indicates that a medium concentration of fibrin provides an optimal microenvironment for high cell viability and osteogenic differentiation of EE-cMSCs (Figure 2C–G). The use of fibrin gel with EE-cMSCs not only maintains the structural integrity necessary for cell proliferation but also simulates the extracellular matrix, facilitating the differentiation of EE-cMSCs into osteoblasts. In the OVCF model, the implantation of EE-cMSCs with fibrin significantly enhanced bone formation by improving the bone microstructure and promoting angiogenesis. Analysis of the trabecular bone around the defect site revealed significant increases in parameters such as BMD, BV/TV, Tb.N, and Tb.Th, while Tb.Sp decreased. Histological staining corroborated these findings by displaying increased new trabecular bone and collagen deposition. Furthermore, the expression of RUNX2, an osteogenic marker critical for osteoblast differentiation and development, and OCN, a marker secreted by osteoblasts, was significantly higher in the EE-cMSCs and EE-cMSC with fibrin groups compared to the OVX group. Notably, the elevated expression of laminin, a marker that facilitates the attachment and migration of endothelial cells to form new blood vessels, confirmed angiogenesis in the EE-cMSCs with fibrin group. High vascularization at the defect site not only supports bone formation but also enhances the vascular network essential for bone regeneration.

This study is not without some limitations. Instead of using human patients with OVCF, we utilized osteoporotic rats with injured tail vertebrae [2,4,67]. OVCFs are common, particularly at the thoracolumbar junction in humans. However, for our study, we chose the coccygeal vertebral body to create bone defects for several reasons. Firstly, the coccygeal vertebrae, despite not bearing direct weight, are rich in metabolically active and rapidly remodeling trabecular bone, making them suitable for studying bone healing and regeneration [68]. Additionally, they share structural and functional similarities with human vertebrae, enhancing their relevance as a model. Their small size and easy accessibility facilitate precise defect creation and analysis, while causing minimal impact on animal mobility and well-being, which is crucial for ethical considerations. The use of coccygeal vertebrae ensures consistent and reproducible results, essential for scientific validity. Moreover, previous studies have established the coccygeal vertebrae as a reliable model for similar research, demonstrating significant bone regeneration [26,69]. These factors collectively support the use of the coccygeal vertebral body for inducing bone defects in osteoporotic vertebral compression fracture studies, aiding in the evaluation of potential treatments.

Our preclinical study highlights EE-cMSCs with fibrin gel as a promising candidate for OVCF treatment. However, cell therapy alone may not suffice to maintain the necessary mechanical properties. For regenerative therapies to be successful, it is crucial to develop new biomaterials combined with highly functional stem cells that provide optimal mechanical properties and pain relief [70,71,72]. Therefore, future research should focus on creating biomaterials integrated with stem cells that can maintain mechanical integrity, enhance osteogenic differentiation, and effectively mitigate inflammatory responses in the clinical management of OVCFs.

## 4. Materials and Methods

### 4.1. Materials

Fibrin gels were formed by mixing fibrinogen from human plasma (Sigma-Aldrich, St. Louis, MO, USA), aprotinin from bovine lung (500 kIU/mL, Sigma-Aldrich, St. Louis, MO, USA), thrombin (2 IU/mL), 10X calcium chloride (Sigma-Aldrich, St. Louis, MO, USA) to achieve a final concentration of 0.8 mM, and 20X phosphate-buffered saline (PBS) to reach a final concentration of 1X. The fibrin gels were prepared at varying fibrinogen concentrations—6.25 mg/mL for the low-concentration gel, 12.5 mg/mL for the mid-concentration gel, and 25 mg/mL for the high-concentration gel [73]. Fibrinogen solution containing aprotinin was polymerized into fibrin gel by applying thrombin mixed with calcium chloride and PBS at 37 °C. Collagen gels were similarly prepared by mixing PureCol^®^ collagen (Advanced Biomatrix, Carlsbad, CA, USA), NaOH (Sigma-Aldrich, St. Louis, MO, USA), and 20X PBS. The collagen gels were formulated with different collagen concentrations—0.625 mg/mL for the low-concentration gel and 1.25 mg/mL for the mid-concentration gel [74]. NaOH was added to achieve a final concentration of 40 mM and 20X PBS to a final concentration of 1X. PureCol^®^ collagen with a pH of 1.9–2.1 was neutralized by mixing with NaOH in PBS to form collagen gel at 37 °C. TissueFill (Cha Meditech, Seongnam-si, Gyeonggi-do, Republic of Korea), a hyaluronic acid (HA) filler, undergoes gelation at 37 °C.

### 4.2. Cell Isolation and Culture

The study received approval from the Institutional Review Board (IRB) of CHA University, School of Medicine (IRB No. GCI-18-34), and was conducted with the written consent of a patient, from whom human fetal skull tissues were obtained following an ectopic pregnancy in 2019. Non-calcified skull tissue in the membranous stage, consisting of a thin cell layer before calcification, was isolated from an 8-week-old ectopic pregnancy fetus. Cells were separated by treating the isolated skull tissue with a collagenase solution at a concentration of 1 mg/mL for 10 min at 37 °C. These EE-cMSCs were obtained through culture dish attachment cell isolation from the fetal skull tissues. EE-cMSCs were cultured in alpha-modified Eagle’s minimum essential medium (α-MEM, Gibco, Thermo Fisher Scientific, Waltham, MA, USA) containing 10% fetal bovine serum (FBS; Gibco, Thermo Fisher Scientific, Waltham, MA, USA), 2 ng/mL basic fibroblast growth factor (bFGF; Gibco, Thermo Fisher Scientific, Waltham, MA, USA), and 25 μg/mL gentamicin (Gibco, Thermo Fisher Scientific, Waltham, MA, USA). The EE-cMSCs were cultured under conditions of 37 °C with a 5% CO_2_ atmosphere, and the medium was changed every 2 to 3 days. When the cells reached more than 80% confluence, they were detached using TrypLE (Gibco, Thermo Fisher Scientific, Waltham, MA, USA) and subcultured. The cultured cells were frozen using CS10 (BioLife Solutions, Bothell, WA, USA) for research use and stored at −180 °C or lower using liquid nitrogen. It was confirmed that the EE-cMSCs maintained their proliferative and differentiation abilities until passage 20, and cells from passage 13 were used in this study. For three-dimensional (3D) osteogenic differentiation, EE-cMSCs were combined with clinically available hydrogels such as atelocollagen gel, fibrin gel, and hyaluronic acid gel. As detailed in Table 1, these hydrogels were prepared to include 5 μg/mL BMP-2 (R&D systems, Minneapolis, MN, USA) to promote osteogenic differentiation. The EE-cMSCs were seeded at a density of 5 × 10^5^ cells per 50 μL of hydrogel in polydimethylsiloxane molds (6 mm diameter) and cultured in α-MEM supplemented with 10% FBS and 25 μg/mL gentamicin for 2 weeks. RAW264.7 cells were used to evaluate osteoclastogenesis induced by RANKL (R&D systems, Minneapolis, MN, USA). These cells were cultured in high-glucose Dulbecco’s Modified Eagle Medium (DMEM) containing 10% FBS and 1% antibiotics. All cells were cultured in incubator at 37 °C with 5% CO_2_, and medium was changed every 2 to 3 days.

### 4.3. Alizarin Red S Staining

The cells were subjected to Alizarin Red S staining to analyze extracellular matrix deposition [75,76]. Cells were fixed in 4% paraformaldehyde, incubated over night at 4 °C, washed with Dulbecco’s PBS (DPBS, Cytiva, Marlborough, MA, USA), and then stained with 1% Alizarin Red S solution (Sigma-Aldrich, St. Louis, MO, USA) for 30 min. To quantify the Alizarin Red S staining, after washing with DBPS, 20% methanol and 10% acetic acid in distilled water were added, and cells were incubated for 15 min to elute the stain. Then, the absorbance of solubilized calcium-bound alizarin red S was measured at 450 nm using a microplate reader (SpectraMax-ID5, Molecular Devices, San Jose, CA, USA).

### 4.4. 3D Cell Culture Mold Fabrication

The three-dimensional (3D) cell culture mold was fabricated using Polydimethylsiloxane (PDMS, Sylgard 184 kit, Dow Corning, Midland, MI, USA) [77]. Sylgard A and B were mixed at a 10:1 ratio according to the manufacturer’s instructions and dispensed into 3.5 cm cell culture dishes. The mixture was poured to a height calculated to ensure that each 6 mm diameter well would hold a total volume of 50 µL (25 µL of EE-cMSCs (5 × 10^5^ cells) and 25 µL of hydrogel) (Figure 2A,B). The molds were cured in a drying oven at 65 °C for 3 h, then cooled and punctured with a 6 mm biopsy punch. The molds were then sterilized with 70% ethanol, washed twice with DPBS (Cytiva, Marlborough, MA, USA), and exposed to UV light for 30 min. Subsequently, 50 µL of the EE-cMSCs and hydrogel mixture was added to each sterilized PDMS mold well. The cell–hydrogel mixtures included all concentrations of hydrogel groups as detailed in Table 1. The samples were then incubated for 30 min at 37 °C to allow the hydrogel to solidify. Afterward, 2 mL of EE-cMSC medium was added to each well, and the cultures were incubated for 24 h at 37 °C and 5% CO_2_, allowing the cells to interact with the surrounding hydrogel matrix in a 3D network that mimics the natural extracellular matrix (ECM).

### 4.5. Cell Viability

The viability of EE-cMSCs in injectable hydrogels was analyzed using the LIVE/DEAD^®^ Viability/Cytotoxicity Kit (Invitrogen, Carlsbad, CA, USA), which contains calcein-AM and ethidium homodimer-1 (EthD-1). This assay distinguishes live cells from dead cells based on cell membrane integrity and esterase activity. The injectable hydrogel experimental groups were prepared by combining EE-cMSCs (5 × 10^5^ cells/25 µL) with different hydrogel formulations (25 µL) to create six distinct groups—fibrin-low, fibrin-mid, fibrin-high, collagen-low, collagen-mid, and TissueFill. These cell–hydrogel mixtures (50 µL) were placed into PDMS mold wells to create a 3D cell culture environment, and after incubating for 24 h at 37 °C, the cell viability assay was performed.

The procedure was performed as follows: The existing medium in the 3D cultures was removed, and the cultures were washed twice with DPBS. According to the manufacturer’s instructions, 5 µL of calcein-AM and 20 µL of EthD-1 were added to 10 mL of fresh culture medium and mixed thoroughly. After washing, 2 mL of this solution was added to each dish and the samples were incubated in the dark at room temperature for 5 min. Finally, the samples were washed twice with EE-cMSC medium, and images were captured using a fluorescence microscope at the appropriate wavelengths (494/515 nm for live cells and 528/617 nm for dead cells) in the presence of the medium. Cell viability was analyzed by quantifying the ratio of live cells to dead cells using FIJI ImageJ software (version 1.54f, National Institutes of Health, Bethesda, MD, USA) based on the captured fluorescence images.

### 4.6. Co-Culture of EE-cMSCs with RAW264.7 Cells

We performed co-culturing of EE-cMSCs and RAW cells using a Transwell system to evaluate the inhibitory effects of EE-cMSCs on osteoclast differentiation under RANKL conditions [78,79]. The experimental setup included the following four distinct groups: 1. Experimental Group 1 (RANKL+, EE-cMSC-): This group was treated with 100 ng/mL RANKL to induce osteoclast differentiation in RAW cells without the presence of EE-cMSCs. 2. Experimental Group 2 (RANKL+, EE-cMSC+): This group was treated with 100 ng/mL RANKL and co-cultured with EE-cMSCs to evaluate the impact of EE-cMSCs on RANKL-induced osteoclast differentiation. 3. Negative Control Group (RANKL-, EE-cMSC-): This group did not receive RANKL treatment and was not co-cultured with EE-cMSCs, serving as a baseline to observe the natural state of RAW cells without osteoclast induction or EE-cMSC influence. 4. Positive Control Group (RANKL-, EE-cMSC+): This group did not receive RANKL treatment but was co-cultured with EE-cMSCs to assess any potential effects of EE-cMSCs on RAW cells in the absence of osteoclast differentiation signals (Figure 1A).

RAW cells (5 × 10^3^ cells/cm^2^) were first seeded into a 12-well plate (Falcon^®^ Cell Culture Insert Companion Plates, Corning Inc., New York, NY, USA) for all groups. After incubating for 24 h, the medium was changed, and RANKL was added at a concentration of 100 ng/mL for the groups with RANKL. Subsequently, Transwell inserts (Falcon^®^ Cell Culture Inserts, pore size 3.0 μm, Corning Inc., New York, NY, USA) were placed on top of the 12-well plate for the RANKL-, EE-cMSC+ group and the RANKL+, EE-cMSC+ group, and EE-cMSCs (1 × 10^4^ cells/cm^2^) were seeded into the inserts. The RANKL-, EE-cMSC- group and the RANKL+, EE-cMSC- group were cultured with RAW cell medium in 12-well plates without cell culture inserts. The four groups were incubated at 37 °C for 4 days, with a medium change on day 2. On the fourth day, TRAP staining, phalloidin staining, and PCR analysis were performed. Additionally, we conducted an experiment under the same group conditions to assess the inhibitory effect of EE-cMSCs on osteoclast differentiation using conditioned medium (Figure S1). The conditioned medium was prepared by sampling the supernatant from the EE-cMSC cultures.

### 4.7. TRAP Staining

TRAP staining was conducted on the fourth day of culturing the cells to assess osteoclast differentiation across the four groups. After removing the Transwell insert, the plates underwent TRAP staining using a kit from TAKARA, JP. The cells were fixed in a fixation solution for one minute and subsequently washed twice with DPBS. To identify the tartrate-resistant enzyme, the fixed cells were stained with an acid phosphatase substrate solution that included sodium tartrate. Following the staining process, the cells were washed three times with sterile distilled water to halt the reaction and were then examined under a microscope (Figure 1B).

### 4.8. Phalloidin Staining

Coverslips were placed in 12-well culture plates, and RAW264.7 cells were seeded into four groups. On the fourth day of culture, phalloidin staining was performed for analysis. The cells were fixed with 4% PFA for one minute and subsequently washed twice with DPBS for 10 min each. Following this, the cells were treated with 0.1% Triton X-100 for 15 min to permeabilize the cell membranes. After two additional 5-min washes with DPBS, the cells were stained with phalloidin Alexa Fluor 555 (1:400, Invitrogen, Carlsbad, CA, USA) in DPBS for 45 min. Subsequently, the cells were washed three times with DPBS for 5 min each. The nuclei were then stained with DAPI (Thermo Fisher Scientific, Waltham, MA, USA), diluted 1:10,000 in DPBS, for 5 min and washed three times with DPBS for 5 min each. The coverslips were carefully removed and mounted on slides using Dako Fluorescent Mounting Medium (Agilent, Santa Clara, CA, USA). The stained specimens were observed using a confocal microscope (Axiovert 200M, Carl Zeiss, Oberkochen, Germany).

### 4.9. RT-PCR and Quantitative PCR

Gene expression analysis of osteoclast markers was conducted on the fourth day in the RAW264.7 cells seeded in a co-culture Transwell system. Additionally, to assess osteogenesis in a 3D culture system, gene expression levels were evaluated on the fourth day in EE-cMSCs mixed with a hydrogel (Fibrin/collagen/TissueFill). Total RNA was extracted using TRIzol reagent (Sigma-Aldrich, St. Louis, MO, USA) and its purity and concentration were measured using a Nanodrop. The Maxime RT PreMix kit (Invitrogen, Carlsbad, CA, USA) was used to transcribe DNase-treated total RNA into cDNA. Subsequently, qRT-PCR was performed using PowerUp™ SYBR™ qPCR Mix (Applied Biosystems, Waltham, MA, USA) on a StepOnePlus Real-Time PCR system (Applied Biosystems, Waltham, MA, USA). Gene expression levels were normalized to the housekeeping gene, glyceraldehyde 3-phosphate dehydrogenase (GAPDH). Primer sequences for the gene of interest used in this study are provided in Table 2.

### 4.10. Animals and OVCF Modeling Procedures

Thirty-three 8-week-old female Sprague–Dawley rats, weighing between 200 and 220 g, were acquired from Orient Bio Inc. (Seongnam-si, Gyeonggi-do, Republic of Korea). These rats underwent a one-week acclimatization period in an environment with a 12/12 h light/dark cycle, at a temperature of 22 ± 1 °C and a relative humidity of 50% ± 1%. During this time, they had unrestricted access to food and water. Following this adaptation period, the animal experiments were conducted in compliance with the guidelines set by the Institutional Animal Care and Use Committee (IACUC) of CHA Bundang Medical Center (IACUC 220190).

The peritoneal area was sterilized using 70% alcohol, and rats were anesthetized with a combination of zolazepam and tiletamine (Zoletil, 50 mg/kg, intraperitoneally, Virbac Laboratories, Carros, France) and xylazine (Rompun, 10 mg/kg, intraperitoneally, Bayer, Seoul, Republic of Korea). For the bilateral ovariectomy, each rat was placed ventral side down, the lumbar hair was removed, and the area was disinfected with 70% alcohol followed by povidone-iodine. A 1 cm midline dorsal incision was made using the dorsolateral skin incision method. The ovarian fat pad was exposed, tied off, and the ovaries were excised. The incisions in the skin and muscle were then sutured and disinfected. Postoperatively, the rats were administered analgesic (Ketoprofen, SCD Pharm. Co., Ltd., Seoul, Republic of Korea) and antibiotic (cefazolin, Chong Kun Dang, Seoul, Republic of Korea) for 3 days. To maintain body temperature, a heating pad was used to keep it at 37 °C.

After 12 weeks of inducing osteoporosis, a coccygeal vertebral bone defect model was established. Under anesthesia, as previously described, a cylindrical defect with a diameter of 2 mm was created in the proximal part of both the seventh and eighth coccygeal vertebrae (Co 7, Co 8) using a trephine drill [27,28]. The rats were then randomized into three groups, namely the injury group (OVX + defect, *n* = 11), the EE-cMSC group (OVX + defect + EE-cMSC (5 × 10^3^ cells/10 µL), *n* = 11), and the EE-cMSC with fibrin gel group (OVX + injury + EE-cMSC (5 × 10^3^ cells/10 µL), *n* = 11). Bone wax was applied to prevent bleeding and stabilize the defect area after the cell injection. From two days before the cell injection until euthanasia, all rats received cyclosporine (100 μg/mL, Cipol-N, Chong Kun Dang, Seoul, Republic of Korea) in their drinking water for immunosuppression. Four weeks after implantation, the rats were euthanized by CO_2_ asphyxiation, and their vertebral bodies were collected for analysis.

### 4.11. Micro-Computed Tomography (μCT) Analysis

Radiographic image acquisition (9 μm pixel) and microstructural reconstruction of coccygeal vertebrae (Co 7, Co 8) were conducted using a μCT system (SkyScan 1172, Bruker^®^, Kontich, Belgium) and NRecon software (version 1.7.3.1; Bruker^®^, Kontich, Belgium) [43]. The regions of interest (ROIs) were categorized into volume of interest (VOI) 1 for bone defects and VOI 2 for the area adjacent to the center of the defect. Key measurements, performed using CTan software (version 1.17.7.2; Bruker^®^, Kontich, Belgium), included bone mineral density (BMD), bone volume to total volume ratio (BV/TV), trabecular number (Tb.N), trabecular thickness (Tb.Th), and trabecular separation (Tb.Sp), in accordance with the manufacturer’s guidelines. VOI 1 was defined as a band of 1.98 mm extending across 70 slices, excluding cortical bone. VOI 2, a subregion near the defect, was analyzed within a 1 mm band extending across 37 slices, excluding the central cavity. Additional segmentation parameters and measurements were utilized to assess trabecular and cortical bone characteristics. 3D reconstructions were generated using CTVox software (version 3.3.0.0; Bruker^®^, Kontich, Belgium) [80].

### 4.12. Histological Analysis

The coccygeal vertebral bone tissues were initially fixed in 4% paraformaldehyde for one week. After fixation, the tissues underwent a two-week decalcification process using Rapid Cal Immuno (BBC Biochemical, Mount Vernon, WA, USA). The samples were then prepared for paraffin embedding and sectioned to a thickness of 10 µm using a Leica microtome. Following this, the sections were dewaxed, rehydrated, and stained with Masson’s trichrome and Hematoxylin and Eosin (H&E staining kit, Abcam, Cambridge, UK) according to established protocols.

### 4.13. Immunohistochemistry

For immunohistochemical analysis, 10 µm paraffin-embedded sections were deparaffinized, rehydrated, and stained with primary antibodies against OCN (8 μg/mL, R&D Systems, Minneapolis, MN, USA), RUNX2 (1:400, Abcam, Cambridge, UK), OPG (1:200, Invitrogen, Carlsbad, CA, USA), RANKL (10 μg/mL, Abcam, Cambridge, UK), and laminin (1:700, Invitrogen, Carlsbad, CA, USA). Antigen retrieval involved heating the sections in a pH 9.0 Tris-EDTA buffer (Biosesang, Yongin-si, Republic of Korea) to 95 °C for 10 min, followed by a 30-min cooling period in the same buffer. After antigen retrieval, sections minwere treated with BLOXALL blocking solution for 10 min to inactivate endogenous peroxidase and alkaline phosphatase. The sections were then washed once with 1X PBS and incubated for 20 min with blocking serum (Vectastain Elite ABC HRP kit, Vector Laboratories, Inc., Newark, CA, USA) to prevent nonspecific binding. Primary antibodies were applied at the recommended dilutions in Blocking Serum. After 24 h of incubation with the primary antibodies, sections were washed with 1X PBS and incubated with a biotinylated secondary antibody (Vectastain Elite ABC HRP kit, Vector Laboratories, Inc., Newark, CA, USA) for 30 min at room temperature. Following another 1X PBS wash, an Avidin–Biotin Complex (ABC) solution (Vectastain Elite ABC HRP kit, Vector Laboratories, Inc., Newark, CA, USA) was applied for 30 min at room temperature. After an additional 1X PBS wash, sections were incubated with DAB substrate (Vectastain Elite ABC HRP kit, Vector Laboratories, Inc., Newark, CA, USA) for 10 min. Finally, the stained sections were washed in tap water, followed by sequential washes with 70%, 80%, 90%, and 99% ethanol, and then twice in xylene before mounting the coverslips using Canada balsam. Detailed specifications of primary and secondary antibodies, including their dilution ratios, are provided in Table S1. Digitally scanned images of all stained sections were captured using a digital slide scanner (Axio Scan.Z1, Carl Zeiss, Oberkochen, Germany). Quantitative assessment of immunohistochemistry was performed using FIJI ImageJ software (version 1.54f, National Institutes of Health, Bethesda, MD, USA) to analyze images of the lesion site. This analysis quantified the proportion of the positively stained area (brown) for osteoblastogenesis markers (OCN, RUNX2, OPG, RANKL) and the basement membrane staining of endothelial cells relative to the total area. Images of stained bone tissue were converted from color to binary, and the threshold was manually adjusted to ensure that only the protein expression regions were visible. Subsequently, the total area of the tissue section and the area of protein staining were measured to calculate the protein-positive area (%) in the image [41,43,44,81].

### 4.14. Statistical Analysis

Data from cytochemical and histological evaluations were processed and analyzed using FIJI ImageJ software (version 1.54f, National Institutes of Health, Bethesda, MD, USA). The results are presented as the mean ± standard error of the mean. Statistical analysis was conducted using one-way ANOVA test, followed by post hoc comparisons with Tukey’s test to identify differences among the groups. Graphical representations of the data and statistical analyses were created using GraphPad Prism (version 8.0.2, GraphPad Software, La Jolla, CA, USA). A *p*-value of less than 0.05 was considered statistically significant. Detailed explanations of the statistical significance and analyses are included in the captions of each figure.

## 5. Conclusions

In this study, we explored the effects of human EE-cMSCs on osteoclastogenesis and osteogenesis in vitro. Our findings demonstrate that EE-cMSCs significantly inhibit osteoclastogenesis while enhancing osteogenesis. In a rat model with osteoporotic vertebral body defects, using EE-cMSCs combined with fibrin gel substantially improved bone regeneration and enhanced bone microarchitecture. These results indicate that EE-cMSCs could significantly improve bone healing and regeneration, underscoring their potential as a therapeutic option for OVCFs. The use of EE-cMSCs alongside advanced delivery systems represents a notable advancement in the management of OVCFs.

## Figures and Tables

**Table 1:** Fibrin and collagen concentrations used for gel synthesis.

	Concentration	Fibrinogen (mg/mL)	Aprotinin (kIU/mL)	Thrombin (IU/mL)	10X CaCl_2_ (mM)	20X PBS (X)
Fibrin gel	Low	6.25	500	2	0.8	1
	Mid	12.5	500	2	0.8	1
	High	25	500	2	0.8	1
	**Concentration**	**Collagen** **(mg/mL)**		**NaOH** **(mM)**	**20X PBS** **(X)**	
Collagen gel	Low	0.625		40	1	
	Mid	1.25		40	1	

**Table 2:** Sequences of primers for quantitative PCR analysis.

Primer	Directions	Sequences
m-RANK	Forward	CACTGGAACTCAGACTGCGA
	Reverse	TTGTTGAGCTGCAAGGGATG
m-CTSK	Forward	GCACCCTTAGTCTTCCGCTC
	Reverse	ACCCACATCCTGCTGTTGAG
m-NFATc1	Forward	CTGCAACAAGCGCAAGTACA
	Reverse	AGGTCCAGAGTGCTATCGGT
m-TRAP	Forward	CACTCCCACCCTGAGATTTGT
	Reverse	CATCGTCTGCACGGTTCTG
h-BSP	Forward	GCTCAGCATTTTGGGAATGGC
	Reverse	CTGCATTGGCTCCAGTGACAC
h-ALP	Forward	CCAAGATCTCCAACATGACT
	Reverse	TCATAGAAGTTGATTACCACAT
h-RUNX2	Forward	CATGTCCCTCGGTATGTCCG
	Reverse	ACTCTGGCTTTGGGAAGAGC

## Data Availability

All datasets used and/or analyzed during the current study are available from the corresponding author on reasonable request.

## Supplementary Materials

The following supporting information can be downloaded at: https://www.mdpi.com/article/10.3390/ijms25158174/s1.

## Abbreviations

OVCFs	osteoporotic vertebral compression fractures
MSCs	mesenchymal stem cells
EE-cMSCs	ectopic embryonic calvaria derived mesenchymal stem cells
bFGF	basic fibroblast growth factor
BMP-2	bone morphogenetic protein-2
RANK	receptor activator of nuclear factor kappa-B
RANKL	receptor activator of nuclear factor kappa-B ligand
NFATc1	nuclear factor of activated T-cells, cytoplasmic 1
CtsK	cathepsin K
TRAP	tartrate-resistant acid phosphatase
RUNX2	runt-related transcription factor 2
OVX	ovariectomized
BMD	bone mineral density
BV/TV	bone volume over total volume
Tb.N	trabecular number
Tb.Th	trabecular thickness
Tb.Sp	trabecular separation
OPG	osteoprotegerin
OCN	osteocalcin
