# Supplementary material for: Promotion of Bone Formation in a Rat Osteoporotic Vertebral Body Defect Model via Suppression of Osteoclastogenesis by Ectopic Embryonic Calvaria Derived Mesenchymal Stem Cells
Source: Int J Mol Sci. 2024 Jul 26;25(15):8174. doi: 10.3390/ijms25158174 (PMC11311643; doi:10.3390/ijms25158174)
Supplement: Supplementary file 1 [file ijms-25-08174-s001.zip › ijms-3091454-supplementary.pdf]

Table S1. Information on the antibodies used in this study.

Antibody	Host	Antigen retrieval	Dilution	Supplier
OCN	Mouse	Tris-EDTA, pH 9.0	8 μg/ml (1:200)	R&D Systems
RUNX2	Rabbit	Tris-EDTA, pH 9.0	1:400	Abcam
OPG	Rabbit	Tris-EDTA, pH 9.0	1:200	Invitrogen
RANKL	Mouse	Tris-EDTA, pH 9.0	10 μg/ml (1:200)	Abcam
laminin	Rabbit	Tris-EDTA, pH 9.0	1:700	Invitrogen

Table S2. Literature review on current trends in molecular biological research on osteogenesis and osteoclastogenesis inhibition mechanisms.

Author	Finding of the Study	Pathway	Treatment Modality
Schiavone ML et al. (2024) [56]	RANKL genetic deficiency inhibits the differentiation of skeletal stem and progenitor cells, and functional blockade reduces osteogenic potential.	RANKL deficiency disrupts SSC differentiation, reducing osteoclastogenesis and osteogenesis.	RANKL genetic deficiency
Boyce BF et al. (2023) [57]	NF-κB signaling is critical for both osteoclast and osteoblast differentiation, affecting bone remodeling processes.	RANKL-induced NF-κB signaling through TRAF6 promotes osteoclastogenesis, while TRAF3 limits it.	NF-κB inhibitors
Liu K et al. (2023) [58]	Atsttrin modulates osteoblast and osteoclast activities through TNFR pathways, impacting bone homeostasis.	TNFR pathway modulation reduces RANKL-induced osteoclast activity and promotes osteoblast differentiation.	Atsttrin
Jin X et al. (2023) [59]	Oridonin inhibits osteoclastogenesis via MAPK/NF-κB and promotes osteoblastogenesis via BMP-2/RUNX2, showing potential for osteoporosis treatment.	RANKL-induced MAPK/NF-κB pathway inhibition reduces osteoclastogenesis, BMP-2/RUNX2 promotes osteoblastogenesis.	Oridonin
Yamanouchi D, Igari K (2023) [48]	Inhibition of Wnt signaling via ICG-001 reduces osteoclastogenic macrophage activation and expression of key markers like TRAP and cathepsin K.	RANKL-induced Wnt signaling inhibition reduces osteoclast differentiation and activity.	ICG-001
Wang J et al. (2023) [60]	RhoA deficiency reduces osteoclast activity and increases bone mass via mTOR-NFATc1 signaling inhibition.	RANKL-induced mTOR-NFATc1 signaling inhibition reduces osteoclast activity and promotes bone mass increase.	RhoA deficiency
Kim J et al. (2023) [61]	UBAP2 knockdown reduces osteoblastogenesis and increases osteoclastogenesis, highlighting its importance in bone homeostasis.	UBAP2 pathway modulation reduces RANKL-induced osteoclastogenesis and promotes osteoblastogenesis.	UBAP2 knockdown
Chen F et al. (2022) [62]	Sr-BCP inhibits osteoclast activity and promotes osteoblast differentiation, enhancing ectopic bone formation compared to BCP.	Sr-BCP enhances osteoblast differentiation while inhibiting RANKL-induced osteoclast activity.	Sr-BCP
Molstad DHH et al. (2020) [49]	Hdac3 deficiency leads to increased responsiveness of osteoclasts to RANKL, resulting in enhanced bone resorption.	Hdac3 modulation enhances osteoclast responsiveness to RANKL, increasing bone resorption.	Hdac3 deficiency
Mattson AM et al. (2019) [63]	PHLPP1 deficiency impairs osteoclast bone resorption activity and enhances bone formation in mice.	PHLPP1 deficiency reduces RANKL-induced osteoclast activity, promoting bone formation.	PHLPP1 deficiency
Cai X et al. (2017) [64]	DOK3 inhibits osteoclastogenesis and promotes osteoblastogenesis, balancing bone remodeling.	DOK3 pathway inhibits RANKL-induced osteoclastogenesis and promotes osteoblast differentiation.	DOK3
Kim JY et al. (2014) [65]	Emodin inhibits osteoclast differentiation and stimulates osteoblast formation, making it a potential treatment for osteoporosis.	Emodin modulates RANKL-induced osteoclastogenesis via pathway inhibition.	Emodin

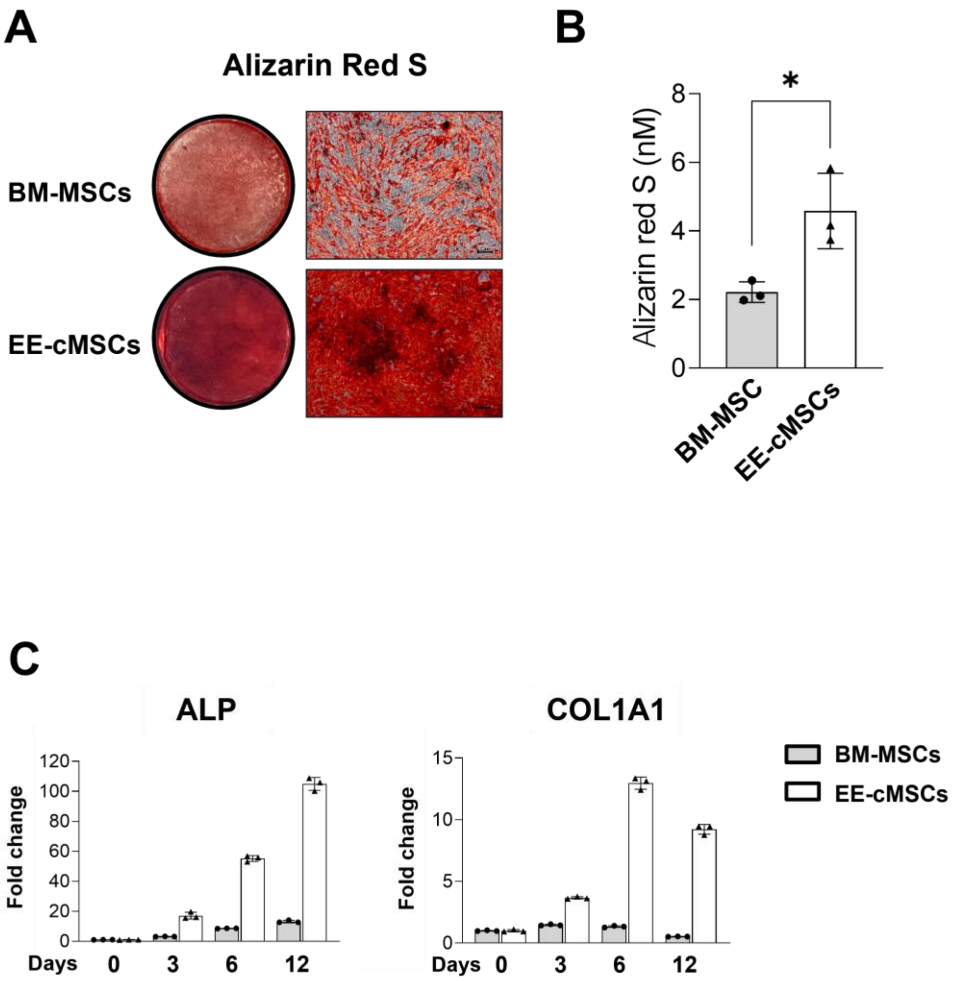

Figure S1. Comparison of osteogenic differentiation between Ectopic Embryonic Calvaria Derived Mesenchymal Stem Cells (EE-cMSCs) and Bone marrow mesenchymal stem cells (BM-MSCs). (A) Microscopical analysis of the osteogenic differentiation potential of EE-cMSCs compared to BM-MSCs. Evaluation with Alizarin Red S was performed on day 21. Scale bar 200 μm. (B) The absorbance value of solubilized Alizarin Red S was higher in EE-cMSCs compared to BM-MSCs. Data are represented as the means ± SD. * denotes statistical significance between the indicated pairs ($p < 0.05$). (C) Gene expression analyses of alkaline phosphatase (ALP) and Collagen, type I, alpha 1 (COL1A1) on day 0, 3, 6, 12, showing higher gene expression in EE-cMSCs compared to BM-MSCs.

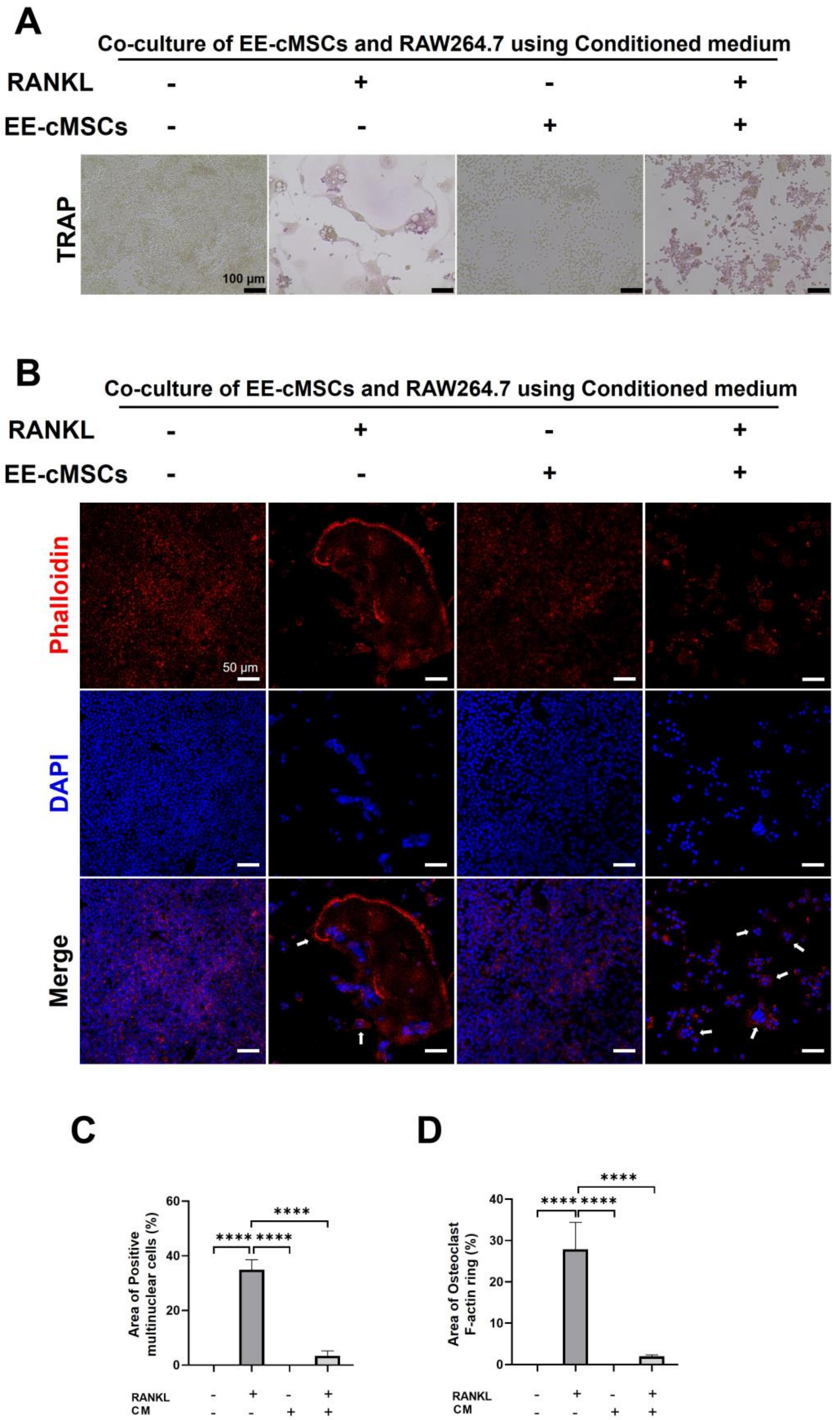

Figure S2. (A) TRAP staining of RAW264.7 cells-derived osteoclast formation in conditioned medium co-culture system. (B) Phalloidin staining of osteoclast actin ring formation in conditioned medium culture system. (C) Quantitative analysis showing the area of positive multinuclear cells. (D) Quantitative analysis showing the area of osteoclast F-actin ring.
